# Feasibility of replacing homemade solutions by commercial products for qualitative fit testing of particulate respirators: a mixed effect logistic regression study

**DOI:** 10.1016/j.mex.2019.05.034

**Published:** 2019-06-01

**Authors:** Anahita Fakherpour, Mehdi Jahangiri, Saeed Yousefinejad, Mozhgan Seif

**Affiliations:** a Department of Occupational Health, Shiraz University of Medical Sciences, Shiraz, 7153675541, Iran; b Department of Epidemiology, School of Health, Shiraz University of Medical Sciences, Shiraz, 7153675541, Iran

**Keywords:** Efficacy of homemade fit solutions as qualitative challenge agents for fit testing of filtering face-piece respirators (FFRs), Qualitative fit test, Challenge agents, Commercial and homemade solutions, Filtering face piece respirators

## Abstract

Qualitative fit testing is mandatory for tight-fitting respirators to ensure that the wearer fitted properly before entering a contaminated workplace. The purpose of this study was to evaluate the homemade solutions as substitution of commercial products for qualitative fit testing of particulate respirators. Two homemade solutions of Bitrex™ and saccharin were made according to the Occupational Safety and Health Administration (OSHA) regulation 29 CFR 1910.134. Threshold Screening Tests (TSTs) of commercial solutions, as well as homemade ones, were conducted on 62 participants in a random order. A placebo was also tested to assure the participants could distinguish its flavorless from other taste of solutions. There were no statistically significant differences between the commercial and homemade solutions representing that participants detected the bitter taste of the Bitrex™ and sweet taste of the saccharin solutions (96.8% vs. 91.9% and 93.5% vs. 83.9%, respectively). Homemade solutions that were stable and haven’t been contained microbial contaminations, could be substituted for commercial products in qualitative fit testing of filtering face-piece respirators (FFRs). Overall, this protocol presents a practical and cost-benefit technique to assess the fit testing of FFRs.

## Graphical abstract

**Figure:**
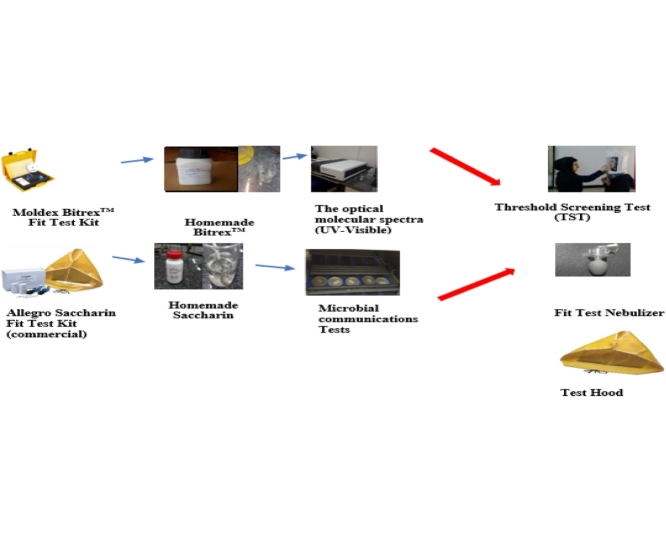

Specifications Table

**Table:** 

Subject Area:	Engineering
More specific subject area:	Occupational health and safety requirements - Respiratory protection program Standards - Fit Testing Procedures
Protocol name:	Efficacy of homemade fit solutions as qualitative challenge agents for fit testing of filtering face-piece respirators (FFRs).
Reagents/tools:	Moldex® Bitrex® Fit Test Kit Part number 0102 (Moldex Co., Culver, Calif.), Allegro® Saccharin Qualitative Fit test Kit Part Number 2040 (Allegro Industries, Paramount, Calif.), Denatonium benzoate)Merck Co., Germany), Sodium saccharin (USP, Sigma-Aldrich Co., USA), Agilent Cary 60 spectrophotometer (Agilent Technologies, Santa Clara, USA), Blood Agar (Merck Co., Germany), Sabouraud Dextrose Agar (Merck Co., Germany), Chloramphenicol (Sigma-Aldrich Co., Canada).
Experimental design:	The commercial and homemade threshold test solutions were prepared according to the OSHA fit test protocol 29 CFR 1910.134. In order to ensure the commercial and homemade solutions would be stable (color, clarity, and not construction of unstable colloids), the chemical parameters (optical molecular spectra) of those solutions were examined on a regular basis using a Spectrophotometer at 25 °C in the wavelength range of 200–800 nm. In order to ensure the commercial and homemade solutions would be safe, the microbial parameters of those solutions were assessed by performing the Blood Agar and Sabouraud Dextrose Agar tests.
Trial registration:	Not applicable
Ethics	Research Ethics Committee of Shiraz University of Medical Sciences: Approval code IR.SUMS.REC.1396.191

Value of the Protocol

**Table:** 

• Valid and enough evidence and documents were obtained from the feasibility of homemade solutions compared to commercial products in order to use in qualitative fit testing of filtering face piece respirators (FFRs). • This protocol could be used as an essential part of the Respiratory Protection Program (RPP) which could promote the culture of conducting the fit testing procedure regularly, then, it provides higher respiratory protection against hazardous contaminants among the workforces. • This protocol is based on the use of homemade solutions for qualitative fit testing of filtering face piece respirators (FFRs) which would be very useful and cost-benefit for fit testing of the respirator wearers during the emergency situations such as an incidence of a pandemic or other infectious disease outbreaks, and surge capacity events due to unavailability and high cost of the commercial fit test kits.

## Description of protocol

Efficiency of air purifying elements as well as face piece appropriateness for users’ face and work environmentare critical factors for provision of optimal respiratory protection [[1], [2], [3]]. Fit testing is an essential part of the Respiratory Protection Program (RPP) that is mandated for tight-fitting respirators based on the respiratory protection standards [[4], [5], [6], [7]]. It is aimed to ensure the respirator fitted adequately into the face [[4], [5], [6], [7], [8], [9]].

There are two types of respirator fit testing including Qualitative fit test (QLFT), based on subjective manner and Quantitative fit test (QNFT), based on the objective manner [4]. High volumes of qualitative fit test (QLFT) solutions are likely expensive and not easily accessible [10]. Therefore, there might have considerable restrictions to do fit test for all subjects, especially during the emergency situations such as an incidence of a pandemic influenza or other respiratory diseases [10]. Notably, this kind of fit test is more widely used [11], because it is simpler to use [10,12], easier to transport [12], faster to perform [10,12,13], and cheaper to set up and maintain [12,13] than quantitative fit test. Also, this method would be very beneficial for the preparation and planning for outbreaks of pandemic diseases and surge capacity events (like disaster or catastrophic), moreover, there is crucial need for sufficient training about fit‐testing procedure of FFRs on the subjects [14].

According to ISO 16975-3 [7], equivalent substances could be used as challenge agents of QLFT which lead to the same results. Remarkably, some qualifications should be considered for selecting a qualitative challenge agent as follows: cost-benefit, availability, safety, suitability for human exposure, and ability to use with any type of approved particular filter [12]. Accordingly, this study was aimed to present a protocol based on homemade solutions for qualitative fit testing of particulate respirators.

### Study design

This was a single-blind, placebo-controlled, and experimental study conducted in Shiraz, Iran.

### Participants

A total of 62 students (37 females and 25 males; mean age: 23.45 ± 4.66 years) of Shiraz University of Medical Sciences were selected using proportional stratified sampling method based on grade. They were evaluated in the Personal Protective Eqiupment (PPE) laboratory of the School of Health.

### Inclusion criteria

The criteria for selecting the participants were included: no getting a cold; no nasal congestion; no allergy to any substance; without cardiovascular or respiratory diseases (for instance, dyspnea or shortness of breath, asthma); rhinoplasty surgery or other factors affecting the taste of solutions. But if any participant got a cold at the time of the study test, the test session was canceled.

### Ethnical aspects

Initially, the participants were briefed on the study purposes and procedures. Then, a written informed consent form was obtained from all the participants before the commencement of the study. Ethical approval for the study was granted by the Research Ethics Committee of Shiraz University of Medical Sciences (approval code IR.SUMS.REC.1396.191). All the procedures were performed in accordance with the protocol approved by the ethics committee.

### Study procedures

Before beginning the study to ensure the threshold solutions haven’t been contaminated, the following measures were taken: Firstly, the solutions have been sterilized. Secondly, the microbial (bacterial and fungal) communications of the solutions were investigated. To do so, the Blood Agar (Merck Co., Germany) was used for bacteria [15] and Sabouraud Dextrose Agar (Merck Co., Germany) containing Chloramphenicol (Sigma-Aldrich Co., Canada) to inhibit bacterial growth, for fungus. After that, the culture media were incubated at 25 °C for 2–7 days. Also, the growth of microbial (bacterial and fungal) species was monitored during incubation (Incubator: 760b-640079, Memmert, Western Germany) [16]. The taste of the solutions was checked routinely. As well, the optical molecular spectra of the threshold check solutions were recorded on a regular basis using Agilent Cary 60 spectrophotometer (Agilent Technologies, Santa Clara, USA) at 25 °C in the wavelength range of 200–800 nm to evaluate the stability of the solutions regarding color, clarity, and not construction of unstable colloids during ten months which were shown in the supplementary file “Figs. S1–S4”.

To be adequately certain that the participants would be able to detect the taste of threshold check solution (lowest possible concentration of qualitative fit test agent), all the participants abstained from eating, chewing gum, and drinking (except for plain water). In the meantime, the placebo solution (distilled water) was tested among the solutions randomly to be sure that participants could distinguish its flavorless from the other taste of solutions. To increase the reliability of the measures, the test conductor asked the participants to drink only plain water. Besides, a 5-minute break allowed to elapse between the test of each solution.

### Threshold check solution preparation

Five different threshold check solutions were made including two commercial and two homemade and a placebo. The commercial solutions were Moldex® Bitrex® Fit Test Kit Part number 0102 (Moldex Co., Culver, Calif.) contained 0.0135% denatonium benzoate, 94.9865% water, and 5% sodium chloride [17] and Allegro® Saccharin Qualitative Fit test Kit Part Number 2040 (Allegro Industries, Paramount, Calif.) contained 1% sodium saccharin and >99% water [18]. Homemade solutions were substantially similar to the commercial products used in the study. The homemade Bitrex™ threshold solution was prepared by dissolving 13.5 mg of denatonium benzoate)Merck Co., Germany) in 100 mL of a 5% (w/v) NaCl solution (Merck Co., Germany) in distilled water. Homemade saccharin threshold solution was developed by dissolving 830 mg of sodium saccharin (USP, Sigma-Aldrich Co., USA) in 100 mL of warm water. Indeed, the concentration of Bitrex™ and saccharin threshold solutions was, respectively, 12.5 and 100 times less than the those of fit test solutions as prescribed in the OSHA standard [4]. All the solutions were decanted into same-appearing bottles and labeled with specific codes by the researchers. Therefore, all the participants were blinded to the contents of each bottle.

### Measures

Threshold Screening Tests (TSTs) were conducted in accordance with the protocol contained in the OSHA respiratory protection standard, regulation 29 CFR 1910.134 [4], The aim of TSTs was to assure the participant being tested could detect the taste of threshold check solutions sufficiently at low levels. A screening test involved placing a hood approximately 12 in. (30.5 cm) in diameter by 14 in. (35.6 cm) tall over a participant’s head, positioning the hood forward (a gap) about 6 in. (15.25 cm) between the participant’s face and hood window, and having a 3.4 in. (1.9 cm) hole in front of the test participant’s nose and mouth area to help ensure the dispersion of the aerosol around the participant’s mouth and accommodate the nebulizer nozzle.

Furthermore, we made periodic checks of the test nebulizers to make sure they were not clogged. Therefore, they held them against a solid dark background to see whether white aerosol cloud appear. Otherwise, we removed the compounds based on the manufacturer’ instructions. Moreover, we regularly rinsed them every 1–2 h to prevent clogging. Besides, we frequently wiped the hood with a paper towel to clean any deposited solution between the tests.

To begin the intervention, the following steps were taken: In the first step, we instructed the participant to place the hood over the head without wearing a respirator, breathe only through their mouth slightly open with tongue extended, and report immediately when he/she could detect the taste (not smell) of the challenge agent. But we didn’t inform the subject about the taste of solutions (bitter, sweet, etc.). In the second step, a taste-screening aerosol was produced in the hood by firmly squeezing the nebulizer bulb. To do so, using the test solutions, we inserted the ten squeezes of the nebulizer bulb into the hole in front of the hood by fully collapsing and expanding the bulb on each squeeze.

In the third step, we asked the participant if he/she could taste the solution; otherwise, we repeated the procedure up to a further two times if required (a total of 30 sprays). Also, the taste threshold was recorded as ten, twenty or thirty regardless of the numbers of squeeze actually completed (either 10, 20 or 30 squeezes). In other words, if the participant could taste the solution between 1–10, 11–20, or 21–30 squeezes, his/her threshold level was categorized into one of the three groups of High, Medium, or Low, respectively. But if the participant was unable to taste the solution after 30 squeezes, he/she was not sensitive to it, then, the threshold test was considered a failure, otherwise, a pass. An online video of threshold screening procedure developed by the manufacturer, Moldex: https://www.youtube.coms/watch?v=xeeBRUC4UZs and Allegro: https://www.youtube.com/watch?v=R8oNMyzS_5Y.

Finally, we took note the collected data such as gender, age, educational level of the participants, threshold solution code, concentration of test solutions (mg/mL), number of sprays, and detection time (sec), threshold level (1, 2, or 3), and test result (pass/fail).

### Data analysis

Since the participants were measured repeatedly. Due to correlated observations, Mixed Effect Logistic Regression (MELR) model including random effects was utilized [19]. Furthermore, the proposed model was adjusted for age and gender. We calculated the Brier score as the Mean square error of taste detection of the solutions [19], accuracy [20], andCohen's kappa value (k) [19] to compare the threshold screening results. A receiver operating characteristic (ROC) curve analysis was computed to assess the detectability of all threshold solutions against placebo (as a reference solution). The area under the ROC curve (AUC) was calculated to evaluate the overall performance of threshold tests of all solutions against placebo [20]. In addition, threshold tests of homemade Bitrex™ and saccharin solutions were compared to those of commercial ones. The statistical significance level was set at p-value ≤0.05. We conducted the analyses with R software.

Table 1 summarizes the descriptive statistics of the results of threshold tests using commercial and homemade solutions. As can be seen, the mean and standard deviation of the number of spray and detection time of commercial Bitrex™ solution was least of all the threshold solutions (5.32 ± 4.18 and 7.96 ± 4.96 (s), respectively). Moreover, a similar proportion of the participants could detect the bitter taste of the commercial and homemade threshold Bitrex™ (96.8% and 91.9%, respectively) and sweet taste of the saccharin solutions (93.50% and 83.90%, respectively).

**Table 1:** Descriptive statistics of threshold screening tests (TSTs) of commercial and homemade solutions.

Solution	Concentration (mg/mL)	Threshold test		Number of spray	Detection time (s)
		Pass N (%)	Fail N (%)	Mean ± SD	Mean ± SD
Placebo	–	44(71)	18(29)	15.68 **±** 8.04	22.08 ± 11.49
Commercial Bitrex™	0. 135	60(96.8)	2(3.2)	5.32 ± 4.18	7.96 ± 4.96
Commercial Saccharin	830	58(93.5)	4(6.5)	7.83 ± 6.43	10.74 ± 10.63
Homemade Bitrex™	0. 135	57(91.9)	5(8.1)	6.23 ± 4.72	9.05 ± 5.37
Homemade Saccharin	830	52(83.9)	10(16.1)	6.92 ± 4.48	11.15 ± 7.19

SD: Standard deviation.

Comparison of the results of TSTs of all solutions against placebo is shown in Table 2. The MELR model revealed statistically significant differences between all the threshold solutions and placebo. It means that the participants coulddetect the bitter taste of (commercial and homemade) Bitrex™ and sweet taste of saccharinthreshold solutions more than flavorless of placebo. The odds ratio of detection of the commercial Bitrex™ solution compared to all threshold solutions was the most (OR = 14.78). Furthermore, homemade saccharin and Bitrex™ solutions had the lowest and highest accuracy score of 81% (101/124) and 0.85% (104/124), respectively. Also, commercial saccharin had the lowest Brier score (0.05).

**Table 2:** Comparison of threshold screening tests (TSTs) of all solutions against placebo by MELR.^a^

Solution		Coefficient (β)	95% CI for β		*OR*^†^	95% CI for OR		Accuracy	Brier Score
			Lower	Upper		Lower	Upper		
Commercial^b^	Bitrex™	2.69*	1.15	4.24	14.78	3.15	69.30	0.84	0.32
	Saccharin	1.94**	0.75	3.13	6.95	2.11	22.91	0.82	0.05
Homemade^b^	Bitrex™	1.68***	0.58	2.79	5.39	1.78	16.34	0.81	0.08
	Saccharin	0.84***	−0.08	1.76	2.32	0.92	5.80	0.85	0.16

a Adjusted for age/gender.

b Homemade and commercial solutions vs. placebo.

* p-value < 0.0001.

** p-value < 0.001.

*** p-value < 0.05.

**Fig. 1:**
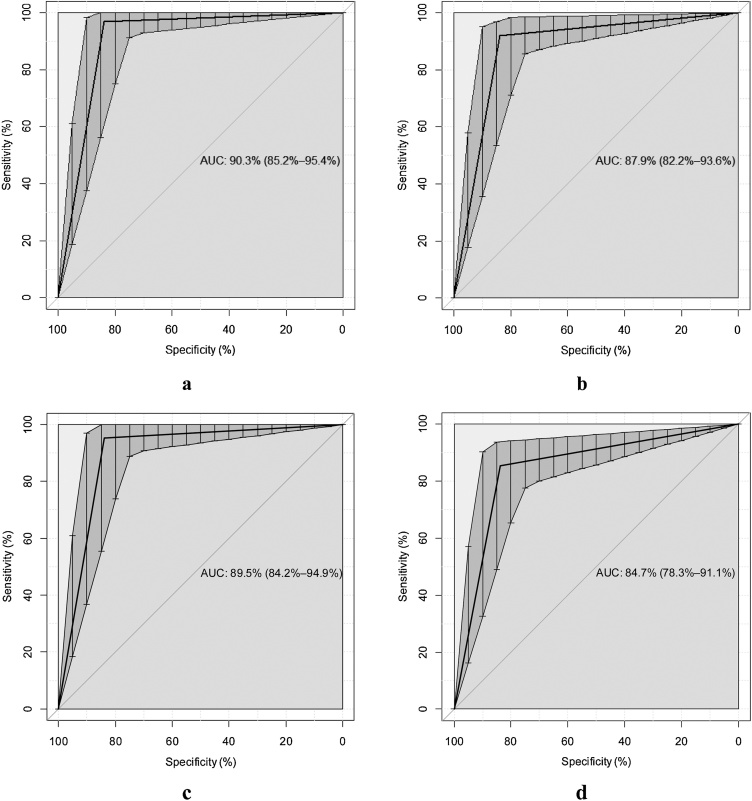
Receiver operating characteristic (ROC) curves for threshold tests of all solutions against placebo: commercial Bitrex™ (a), homemade Bitrex™ (b), commercial saccharin (c), and homemade saccharin (d).

Fig. 1 shows the receiver operating characteristic (ROC) curves for threshold tests of commercial and homemade solutions against placebo that represents the similar performance of commercial and homemade threshold solutions.

The results of the threshold tests of homemade solutions compared to commercial products are summarized in Table 3. There were no statistically significant differences between the results of the homemade and commercial solutions. In other words, Most of the participants coulddetect the taste of homemade and commercial threshold solutions. They could taste not only the commercial solutions but also the homemade ones. Accordingly, homemade solutions could be made locally with equal efficacy to that of commercial products.

**Table 3:** Comparison of threshold screening tests (TSTs) of homemade solutions against commercial products by MELR.^a^

Homemade Solution	Coefficient (β)	95% CI for Coefficient		*OR*^†^	95% CI for OR		Kappa (k)	95% CI for Kappa	
		Lower	Upper		Lower	Upper		Lower	Upper
Bitrex™^b^	−1.0	−2.71	0.70	0.37	0.07	2.02	0.88	0.79	0.97
Saccharin^b^	−1.24	−2.57	0.10	0.29	0.08	1.10	0.83	0.74	0.93

*p-value < 0.05.

a Adjusted for age/gender.

b Homemade solution vs. commercial one.

Among threshold solutions, the commercial Bitrex™ solution was tasted by most of the participants. On the other hand, the odds of taste detection of commercial Bitrex™ and saccharin was, respectively, 14.8 and 6.9 times the odds of placebo. Whilethe odds of taste detection of homemade Bitrex™ and saccharin were 5.40 and 2.30 times the odds of placebo. Likewise, the odds of detection of commercial Bitrex™ and saccharin solutions was, respectively, almost 2.70 and 3.40 times the odds of detection of homemade ones, respectively. Additionally, significant agreements between the test results of homemade and commercial solutions of Bitrex™ and saccharin were found (k = 0.88 and 0.83, respectively) that emphasizes the similar efficacy of homemade solutions to that of commercial products. This finding is consistent with the study conducted by Mitchell et al. [10] that expresses the homemade solutions could be used as the substitution of commercial products for qualitative fit testing of respirators.

Particularly, when commercial solutions would be unavailable or too expensive, we can localize it by lower cost and time-consuming in emergency situations such as an incidence of an influenza pandemic or other respiratory diseases. Besides, if it is impossible for an organization to provide the qualitative or quantitative fit testing equipment, it is the best choice to make the fit test solutions locally to consider the fit testing requirements.

More noticeably, whether commercial or homemade Bitrex™ was tasted more than that of saccharin (96.80% vs. 91.90% for Bitrex™ and 93.5% vs. 83.90% for saccharin, respectively). This is consistent with the findings of McKay et al [14] and Mullins et al. [12] stated that Bitrex™ might be detected better than saccharin. Previous study conducted by Marsh et al., showed that only 4% of the subjects could not detect the saccharin threshold solutions after 30 squeezes of the nebulizer into hood [16]. Thus, the mean and standard deviation of sprays' number of Bitrex™ (13.5 mg/mL) and saccharin (830 mg/mL) solutions were 5.32 and 7.83 for commercial solutions, 6.23 and 6.92 for homemade solutions. However, the number of spray to detection of commercial Bitrex™ (22.5 mg/mL) and saccharin (830 mg/mL) solutions in the study conducted by McKay et al. [14], was 1.35 and 2.38, respectively. It seems that because of a13-fold increase at the concentration of Bitrex™ threshold solution, the numbers of sprays to detection were reduced.

It should be noted that various studies performed on qualitative fit tests [[21], [22], [23], [24], [25]]. Janssen et al. reported that the controlled negative pressure (CNP) was the most conservative fit test followed by Bitrex™ fit testing [17]. Other researches presented that the bitter taste of Bitrex™ aerosol was characterized by most of the study participants during the threshold test [[25], [26], [27]]. Niemandt et al. stated that 4 out of the 24 participants were excluded from the fit test due to facial hair, a blocked nose, claustrophobia, and failing the threshold test [28].

We might be concluded from the previous studies that using homemade solutions as qualitative fit test agents could be a cost-benefit technique, specifically, during the pandemic or other respiratory infection diseases or surge capacity events in which feasibility of doing fit tests for large numbers of health care staff or accessibly to the commercial fit test solutions or would be impossible. One of the vital actions for emergency preparedness planning, it is essential to make locally fit test solutions for emergency situations.

Another one, it also required to mobilize majority of the health care professionals, staff, students, volunteers, as well as, retired and provide adequate training regarding respirator fit testing [14].

Interestingly, the Cost-Benefit Analysis (CBA) of the current study indicates that the cost and time required to access homemade solutions were remarkably less than commercial ones. In the other means, the overall cost of homemade Bitrex™ solution was 30 times (96.7%) lower than that of commercial Bitrex™. Additionally, the required time for preparation of homemade Bitrex™ fit test solution was 10 times lower than that of commercial Bitrex™. Considerably, the overall cost of homemade saccharin fit test solution was 20 times (96.7%) lower than that of commercial saccharin. Additionally, the required time for preparation of homemade saccharin fit test solution was 10 times lower than that of commercial saccharin.

**Fig. 2:**
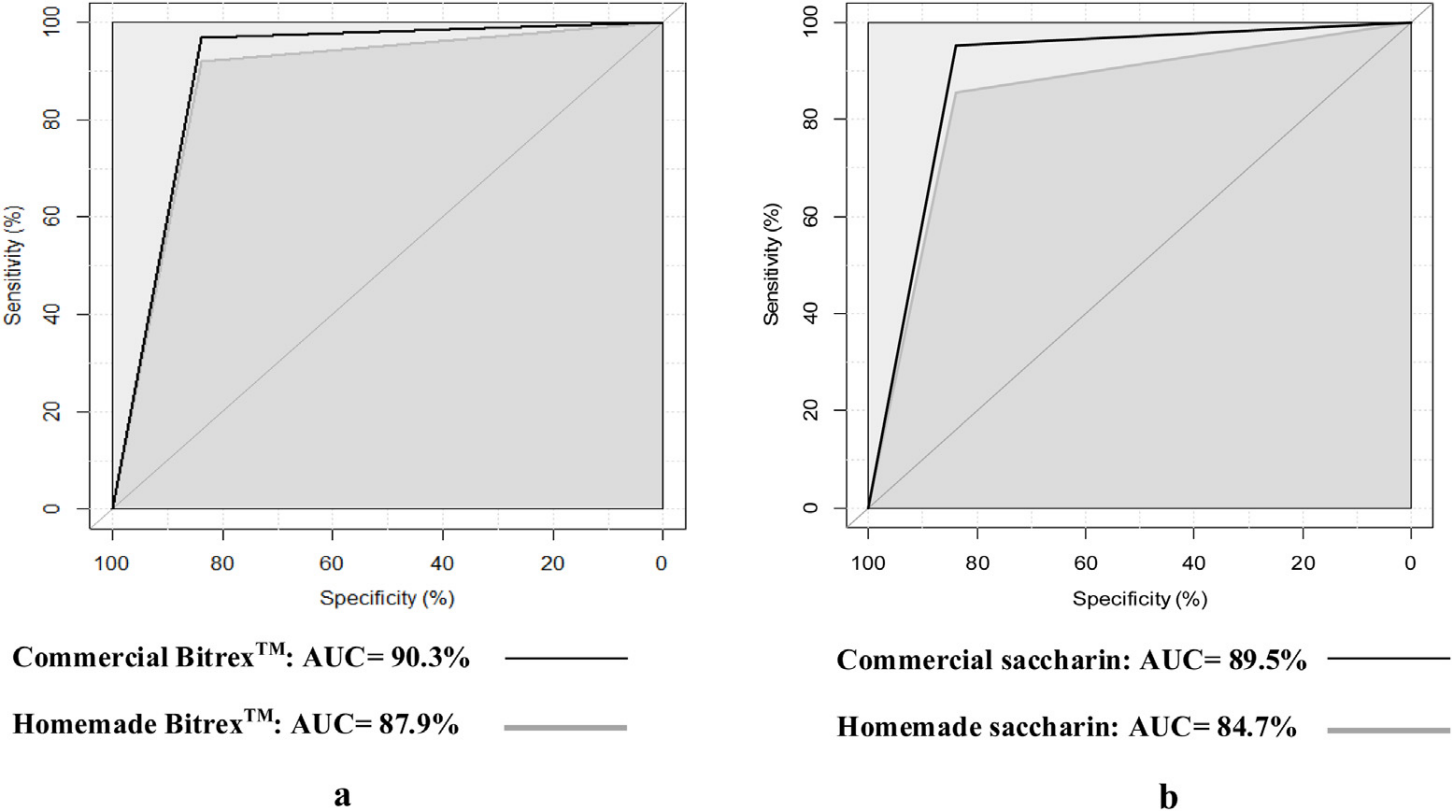
Comparison of Area Under the Roc Curves (AUC) for Threshold Tests of Commercial and Homemade Solutions: Bitrex™ (A) and Saccharin (B).

Fig. 2 compares the area under the ROC curves (AUC) for commercial and homemade Bitrex™ (a) and saccharin (b) threshold tests to determine the overall performance of commercial and homemade solutions against placebo. Consequently, no significant differences were observed between commercial and homemade threshold tests (p-value = 0.26 and 0.29, for Bitrex™ and saccharin, respectively) that highlights the participants correctly detected the taste of both commercial and homemade solutions.

## Conclusion

The results of our study suggest that threshold check solutions could be made efficiently like commercial products and used as qualitative challenge agents. Considerably, the threshold solutions have been safe for microbial communications. Moreover, some physicochemical properties of the solutions (such as color, taste, clearance) were checked which have not been changed for a long time. Nonetheless, the following precautions must be taken: making solutions according to exact concentrations stated in the OSHA protocol [4] on the basis of safety principles, uniformly squeezing the nebulizer bulb by the assessor, repeatedly checking the nebulizer or hood to make certain they weren’t clogged or damaged, and having no allergy to any substance. Additionally, this study was conducted on a small group of students to assess their subjective response to threshold check solutions. As a result, it was highly depended on the accuracy of the subjects’ taste detection.

## Conflict of interest

The authors declare that there are no conflicts of interest.

## Author’s contribution

Data designation: Mehdi Jahangiri, Anahita Fakherpour.

Project administration: Anahita Fakherpour.

Formal analysis: Mozhgan Seif.

Validation: Mehdi Jahangiri, Saeed Yousefinejad.

Writing - review & editing: Mehdi Jahangiri, Anahita Fakherpour.
