# Supplementary material for: Feasibility of replacing homemade solutions by commercial products for qualitative fit testing of particulate respirators: a mixed effect logistic regression study
Source: MethodsX. 2019 Jun 1;6:1313–22. doi: 10.1016/j.mex.2019.05.034 (PMC6558090; doi:10.1016/j.mex.2019.05.034)
Supplement: Supplementary file 1 [file mmc1.docx]

**Supplementary material**

Figure 1-1-S1

Figure 2-1-S2

Figure 3-1-S3

Figure 4-1-S4

**Additional information**

**Description of protocol**

Fit testing is an essential part of the Respiratory Protection Program ‎‎(RPP) that is mandated for tight-fitting respirators [[1-4](#_ENREF_1)]. It is aimed to ensure the respirator fitted adequately into face [[4](#_ENREF_4), [5](#_ENREF_5)]. In recent researches by Jahangiri et al. [[6](#_ENREF_6), [7](#_ENREF_7)], the lowest performance in the implementation of RPP ‎was related to fit testing in Iranian industries and hospitals. There are two types of respirator fit testing: Qualitative fit test and Quantitative fit test [[4](#_ENREF_4)]. QNFT decreases the test subjectivity and provides a fit factor using an instrument [[8-10](#_ENREF_8)].

QLFT relies on a subject’s olfactory or taste response ‎to a challenge agent during a series of simulated work ‎exercises while wearing a respirator. It uses three common challenge agents including isoamyl ‎acetate; saccharin; and Bitrex^TM^.‎ Isoamyl ‎acetate is used for fit testing of respirators ‎equipped with organic vapor cartridges. Saccharin and Bitrex^TM^ are fit test agents of ‎particulate respirators [[4](#_ENREF_4)]. ‎Bitrex^TM^ is the most common challenge ‎agent‎ and trade name of denatonium benzoate ‎(‎‏Smith Ltd., Montvale, NJ‏ ‏McFarland‏‎)‎‏‎, commonly used as a denaturant in commercial products [[11](#_ENREF_11), [12](#_ENREF_12)] and known as the most bitter substance [[11](#_ENREF_11), [13](#_ENREF_13)].

QLFT depends on the wearers' reaction, besides‎, high volume of fit test solutions are likely ‎expensive and not easily accessible. Therefore, there might have considerable restrictions to do fit test for all subjects‎ [[10](#_ENREF_10)], as the incidence of 2009 pandemic influenza H1N1 in Tasmania‏ ‏[[14](#_ENREF_14)]. However, this kind of fit test is more widely used [[15](#_ENREF_15)] , because it’s simpler and cheaper to perform [[8](#_ENREF_8), [14](#_ENREF_14), [16](#_ENREF_16)] than QNFT. According to ISO ‎16975-3 [[2](#_ENREF_2)], ‎equivalent substances could be used as challenge agents which lead to the same results. Accordingly, this study was aimed to present a protocol based on homemade solutions for qualitative fit testing ‎of ‎particulate respirators. ‎
